# Predictors of HIV/AIDS comprehensive knowledge and acceptance attitude towards people living with HIV/AIDS among unmarried young females in Uganda: a cross-sectional study

**DOI:** 10.1186/s12905-021-01176-w

**Published:** 2021-01-26

**Authors:** Tesfaldet Mekonnen Estifanos, Chen Hui, Afewerki Weldezgi Tesfai, Mekonnen Estifanos Teklu, Matiwos Araya Ghebrehiwet, Kidane Siele Embaye, Amanuel Kidane Andegiorgish

**Affiliations:** 1 Department of Maternal, Child and Adolescent Health, School of Public Health, Tongji Medical College, Huazhong University of Science and Technology, Wuhan, People’s Republic of China; 2 Department of Statistics, College of Science, Eritrea Institute of Technology, Mai Nefhi, Eritrea; 3 Department of Educational Administration, College of Education, Asmara, Eritrea; 4 Department of Pathology, School of Basic Medicine, Tongji Medical College, Huazhong University of Science and Technology, Wuhan, People’s Republic of China; 5 Department of Epidemiology and Biostatistics, School of Public Health, Xi’an Jiaotong University Health Science Center, Xi’an, People’s Republic of China

**Keywords:** HIV/AIDS, Unmarried women, Comprehensive knowledge, Acceptance attitude, Uganda

## Abstract

**Background:**

Youth in general and young females, in particular, remain at the center of HIV/AIDS epidemic. To avoid and prevent HIV infection, comprehensive knowledge as well as correct understanding of transmission and prevention strategies are crucial. Thus, the aim of this study is to explore the predictors of comprehensive knowledge on HIV/AIDS and accepting attitude towards PLWHIV.

**Methods:**

A cross-sectional study was conducted using data from the 2016 Uganda Demographic Health Survey. A two-stage probability sampling method was applied and data were collected using a standard questionnaire. Of the total 8674 women aged 15–49 years, 1971 eligible women aged 15–24 years were included in this analysis. Data analysis was done using SPSS version 23. A Chi-square test followed by logistic regression analysis was used to explore the relationship between specific explanatory variables and outcome variables. The results were reported using odds ratios with 95% confidence interval. *P* value less than 0.05 was considered as statistically significant.

**Results:**

Overall, 99.3% of the unmarried women aged 15–24 years were aware of HIV/AIDS, but only 51.9% had comprehensive knowledge on HIV/AIDS. Around 70% of the respondents were aware that "using condoms every time when having sex" and "having only one faithful uninfected partner" can prevent HIV transmission. About 68% of the unmarried women rejected at least two common local misconceptions about HIV/AIDS. An alarmingly small (20.6%) proportion of the respondents had a positive acceptance attitude towards PLWHIV. All variables were significantly associated with having comprehensive knowledge on HIV/AIDS in the unadjusted logistic regression analysis. After adjustment, older age (20–24 years), being educated, wealthier, and ever been tested for HIV/AIDS became predictors of adequate comprehensive HIV/AIDS knowledge. Moreover, respondents with adequate comprehensive knowledge of HIV/AIDS were more likely (OR 1.64, 95% CI 1.30–2.08) to have a positive acceptance attitude towards PLWHIV than their counterparts.

**Conclusion:**

Our study demonstrated a remarkably high level of awareness about HIV/AIDS among study participants, but the knowledge and positive acceptance attitude towards PLWHIV were not encouraging. Thus, endeavors to expand and strengthen educational campaigns on HIV/AIDS in communities, health facilities, and schools are highly recommended. Attention should particularly focus on young-aged and disadvantaged women with low educational level, poor socioeconomic status and those who have never been tested for HIV/AIDS.

## Background

Globally, HIV/AIDS remains a serious public health threat with around 40 million people living with the virus in 2018, of whom about 21% were unaware of their status [1]. Existing data indicate that young females constitute about 20% of the total adult population in terms of new HIV infection. Even though the incidence of HIV/AIDS infections fell by 36% between 2000 and 2017 [2], the reduction is far from the desired target of less than 500,000 new cases by 2020. This calls for a global need to increase the rate at which the world reduces its rate of infections [3]. According to UNAIDS 2020 report, there were 1.7 million new infections and 690,000 AIDS-related deaths in the year 2019 [4]. The global prevalence of HIV/AIDS among women aged 15–24 years was 15%, of whom 80% reside in sub-Saharan Africa (SSA). The SSA is regarded as a high-prevalence setting where more than 70% of all new HIV infections occur [2, 3, 5, 6], and young women are disproportionately impacted [7]. The primary route of HIV transmission in the SSA is believed to be unsafe sexual practice [8]. Three in four new HIV infections in this region occur among girls aged 15–19 years [3]. Likewise, data from UNAIDs 2020 indicated that adolescent women aged 15–24 years have been at higher risk of new HIV infection compared to their male counterpart (24% vs 9%, respectively) [4].

In Uganda, the overall prevalence of HIV infections was 18% in 1992 but declined to 6.4% in 2004. Irrespective of the considerable achievement in lowering new HIV infection, and lessening of AIDS-related deaths, the country continues to have a high (7.3%) prevalence of the infection as of 2011 [9]. The escalation in prevalence is attributed to the ceaseless spread of the virus coupled with the heightened longevity of individuals having the disease [9]. Adolescent girls and young women aged 15–24 years in Uganda are particularly at high risk compared to their male counterparts with a prevalence of 3.2% and 1.9%, correspondingly [9, 10]. Furthermore, a slow rate of decline in new HIV infection was reported in Uganda that poses doubt as to whether Uganda is capable of meeting the fast track targets including: an overall goal of reducing the number of new infections, a goal of reaching zero new infections, with zero HIV/AIDS related deaths and zero discrimination [9, 11]. Existing evidence suggests that high-risk sexual behaviors, low knowledge of one’s HIV serostatus, low level of knowledge and awareness about HIV, and stigmatization of people living with HIV (PLWHIV) are the key drivers of HIV incidence in Uganda [9, 10, 12, 13].

Many previous studies have concluded that increasing comprehensive knowledge and positive attitude are important intervention measures in the prevention of HIV transmission [14–17]. Other studies have demonstrated that young women possess poor comprehensive knowledge about HIV/AIDS compared to their male counterparts despite the elevated level of awareness [14, 18–20]. This indicates that youths in general and young females, in particular, remain at the center of the epidemic [8, 20]. Therefore, the best remedial measures to mitigate the future courses of the epidemic in Uganda could be through enhancing knowledge and attitude of people at younger age.

Although the significance of gathering accurate data on knowledge about HIV/AIDS and level of awareness is universally acknowledged, there is paucity of research addressing the predicting factors of young women’s comprehensive knowledge about HIV/AIDS and their acceptance attitude towards PLWHIV in Uganda. Thus, evaluating the predictors of comprehensive knowledge on HIV/AIDS and attitude towards PLWHIV is vital. In the present study, data were obtained from the 2016 Uganda Demographic and Health Survey (UDHS) of unmarried women aged 15–24 years. Our findings provide valuable insights into the identification of various factors that determine the comprehensive knowledge of HIV/AIDS and acceptance attitude in PLWHIV, and thereby highlight the need to implement a timely intervention among high risk groups.

## Methods

### Study design

A nationally representative cross-sectional study using a stratified two-stage cluster sampling design was used. The design used for data collection in DHS is detailed and published elsewhere [21–23].

### Data source

This study used data from the 2016 Uganda Demographic and Health Survey (UDHS-2016) [24], a nationally representative household cluster survey, that contains data on various indicators of health including HIV/AIDS. It is conducted every five years in low and middle-income countries with a well-defined method of data collection [21, 25]. HIV/AIDS knowledge and attitude-related variables were sorted out and re-categorized based on survey indicators for HIV/AIDS from the MEASURE DHS online tools database [23, 24].

### Study population

A total sample of 8674 women aged 15–49 years were considered during the survey. In this study, a sample of 2019 (unweighted sample) unmarried young women aged 15–24 was abstracted out of the total.

### Data collection

All women of reproductive age (15–49 years) from the selected households were interviewed. During data collection, trained interviewers visited attendants on a house-to-house basis using a standard questionnaire in order to collect an internationally agreed, comparable and homogeneous data [26, 27].

### Independent variables

Socio-demographic details of the study participants were incorporated in the analysis to indicate factors for an achievable intervention and to act as control variables in the analytical models. These variables included: age, religion, educational level, residence, wealth index, ever been tested for HIV and exposure to media. All of them were extracted as they emerged in the data files of DHS. The age of the respondents was grouped into seven categories with a 5-year difference. However, in this study, the first two age categories (15–19 and 20–24 years) were selected. Religion of the participants was re-grouped into five categories: Catholic, Protestant, Muslim, Pentecostal, and Others. Four categories of educational levels: no education, primary, secondary and higher were considered. The place where respondents reside was labeled as either rural or urban. Wealth status of the subjects was also classified into five groups namely; poorest, poorer, middle, richer and richest. Additionally, participants were asked about the frequency of reading newspapers/magazines, listening to radio and watching TV. These composite variables were re-categorized as “not at all” and “at least once a week”. Comprehensive knowledge was considered as a predictive factor for attitude towards PLWHIV and the details of this parameter are fully described in the dependent variable section below.

### Dependent variables

Several validated and reliable questionnaires were employed in the UDHS, one of which was particularly designed for women aged 15–49 years. Questions pertaining to participants’ HIV/AIDS knowledge and attitude toward PLWHIV were included in the questionnaire. We established two binary dependent variables (HIV/AIDS comprehensive knowledge and accepting attitude toward PLWHIV) depending on the DHS measure online tools for HIV/AIDS survey indicator database [23, 24, 26] and previous studies elsewhere [14, 21, 28]. Having comprehensive knowledge about HIV/AIDS is regarded as knowing HIV prevention methods (using a condom and restricting sex partners to one uninfected faithful partner), being aware that any healthy-looking person can have the virus, and rejecting two most common local misconceptions (i.e. HIV can be transmitted through mosquito bites, and by witchcraft or supernatural means). All questions had binary “yes” or “no” response. Those who had comprehensive knowledge were coded “1”, otherwise “0”. Similarly, questions regarding accepting attitude towards PLWHIV were sorted out and re-categorized. If the respondents answered “yes’ at least for the first three of four questions, they were considered as having a positive accepting attitude, and else as having a negative accepting attitude. The questions were: (1) are you willing to care for a sick relative having HIV/AIDS?; (2) can you buy fresh vegetables from a market vendor who is known to have HIV/AIDS?; (3) do you agree that a female teacher who has the virus but is not sick should be allowed to continue teaching?; (4) do you agree the status of any HIV positive family member should remain secret?

### Statistical analysis

Data were analyzed using SPSS version 23, and sampling weights was applied in all analyses by adjusting the sampling design (clustering and stratification) with individual women sample weight divided by one million (V005/1,000,000) as per the DHS’ commands. Descriptive statistics was used to express the characteristics of respondents, their knowledge about HIV/AIDS, and their attitude towards PLWHIV. Frequencies and percentages were presented using tables. The relationship between the outcomes (HIV/AIDS comprehensive knowledge and acceptance attitude) and explanatory variables was tested using the Pearson's Chi square test. Variables which showed significant association (*P* < 0.05) were retained for further multivariable logistic regression analysis. Adjusted multivariable logistic regression analysis was applied to examine the strength of associations between socio-demographic characteristics and the outcome variables. Adjusted Odds Ratios (aORs) with 95% confidence interval (95% CI) were used to report the results of multivariable analyses. *P* value < 0.05 was considered as statistically significant.

### Ethical concerns

The Uganda DHS survey was approved by the Uganda health research Council and ICF Macro Institutional Review Board, in Calverton Maryland, USA. Respondents provided written informed consent for the survey. Participants’ information was handled confidential by deleting their identifiers. The raw data for the present study was obtained after accessibility permission was granted by ICF Macro Inc. in Calverton, Maryland, USA [27].

## Results

There were 1,971 women included in the final analysis after accounting for the sample weights corresponding to the target women. Table 1 illustrates the frequency distribution of the study participants. Majority (80.2%) of the respondents were within the age group of 15–19 years and a higher percentage (74.8%) of them were from rural areas. A small fraction (2.2%) of the participants did not attend school at all. More than half (53.8%) of the respondents had never been tested for HIV, whereas 91.5% had access to mass media at least once a week.

**Table 1:** Descriptive characteristics of the study population (n = 1971)

Variables	Categories	Number (n)^a^	Percentage (%)
Age (years)	15–19	1582	80.2
	20–24	289	19.8
Religion	Catholic	779	39.5
	Protestant	612	31.0
	Muslim	244	12.4
	Pentecostal	283	14.4
	Others	53	2.7
Educational level	No Education	44	2.2
	Primary	1070	54.3
	Secondary	728	36.9
	Higher	130	6.6
Place of residence	Urban	496	25.2
	Rural	1475	74.8
Wealth index	Poorest	220	11.2
	Poorer	269	13.7
	Middle	350	17.8
	Rich	467	23.7
	Richest	664	33.7
Ever been tested for HIV/AIDS	No	1060	53.8
	Yes	912	46.2
Mass media (frequency of listening to radio/watching TV/reading newspaper or magazine))	Not at all	168	8.5
	At least once a week	1799	91.5
HIV/AIDS awareness	No	13	0.7
	Yes	1958	99.3
HIV/AIDS comprehensive knowledge	Do not have	940	48.1
	Have	1015	51.9
Acceptance attitude towards PLWHIV	Do not have	1553	79.4
	Have	403	20.6

^a ^Missing values are excluded

Almost all (99.3%) young women in this study reported that they had ever heard of HIV/AIDS, but only less than half (48.1%) of the them had comprehensive knowledge about HIV/AIDS (Table 1). Less than a quarter of young unmarried women aged 15–24 (20.6%) had an overall positive acceptance attitude towards PLWHIV.

The distribution of correct responses about HIV/AIDS based on respondents’ sociodemographic characteristics is described in Table 2.

**Table 2:** Percentage distribution of women’s HIV/AIDS knowledge and their Socio-demographic variables

Variables	Women who knew prevention methods			Women who knew a healthy looking person can have HIV/AIDS	Rejecting common local misconceptions				Women with comprehensive knowledge
	Using condom every time having sex	Having only one faithful uninfected partner	Knew both prevention methods		Can get HIV from mosquito bites	Can get HIV by sharing food	Can get HIV by witchcraft or supernatural power	Reject at least two common local misconceptions	
Total	76.6	86.2	70.4	83.3	66.9	80.5	87.2	68.1	51.9
*Age (years)*									
15–19	74.9	85.3	68.4	81.6	66.0	80.2	86.2	66.2	48.9
20–24	83.5	90.0	78.5	90.2	70.2	81.5	91.0	75.9	63.8
*Religion*									
Catholic	75.8	86.2	69.5	80.7	66.2	77.8	85.0	64.6	47.1
Protestant	76.4	88.4	71.5	82.6	68.6	85.1	88.1	70.6	54.7
Muslim	79.2	80.0	69.0	89.3	64.8	79.5	91.4	76.6	52.9
Pentecostal	78.0	87.5	72.3	87.2	65.2	76.6	87.2	64.9	53.8
Others	69.8	84.6	67.3	83.0	75.0	92.5	90.6	67.9	57.8
*Educational level*									
None	33.3	71.4	33.3	48.8	44.2	59.5	61.9	28.6	13.8
Primary	73.0	83.3	65.6	78.3	58.0	73.8	82.7	57.6	40.5
Secondary	82.5	91.5	78.8	89.9	77.5	89.8	94.1	81.8	65.5
Higher	87.6	85.4	74.8	97.7	86.9	90.0	93.1	90.0	74.8
*Place of residence*									
Urban	83.0	89.0	77.7	92.5	72.7	82.6	91.9	79.7	62.7
Rural	74.5	85.2	67.9	80.2	64.9	79.7	85.6	64.2	44.5
*Wealth index*									
Poorest	63.9	79.5	58.4	70.9	53.6	74.0	73.2	45.2	32.0
Poorer	66.4	86.5	63.4	74.2	61.4	79.1	81.3	57.7	41.9
Middle	79.5	85.1	69.6	80.9	63.5	78.3	90.4	65.1	50.4
Richer	80.4	85.8	74.8	84.5	66.0	77.6	87.7	70.2	54.4
Richest	80.8	89.1	73.8	91.2	75.8	86.3	92.3	80.1	61.6
*Ever been tested for HIV/AIDS*									
No	70.3	83.0	63.5	79.1	62.9	77.3	84.1	63.3	45.0
Yes	83.8	89.9	78.3	88.2	71.4	84.1	90.7	73.7	59.8
*Mass media (frequency of listening to radio/watching TV/reading newspaper or magazine)*									
Not at all	73.5	72.9	60.8	71.7	57.8	73.5	81.9	48.8	38.6
At least once a week	76.9	87.4	71.3	84.4	67.7	81.1	87.6	69.8	53.2

Others in religion: Seven Day Adventist, Baptist, Eastern Orthodox Christian, Bahai Faith, Jehovah’s Witnesses, Traditional African Religion, Presbyterian, Hindus, Jews, and Buddhists

Accordingly, 86.2% of the participants mentioned that HIV/AIDS transmission can be prevented through “having only one faithful uninfected partner”, and 76.6% of them by “using condom every time one has sex”. Around 70% of the participants mentioned both the above methods as the best practices of prevention. Concerning the common local misconceptions about HIV transmission, two-third (66.9%) of the respondents rejected the belief that “anyone can get HIV from mosquito bites”, 80.5% disagreed with the notion that one “can get HIV by sharing food”, and 87.2% disagreed with the idea that one “can get HIV by witchcraft”.

Table 3 describes the percentage distribution of women with an accepting attitude towards PLWHIV for the different attitude questions. As per the analysis, majority (86.2%) of the study respondents were willing to care for a relative who was diagnosed with HIV/AIDS and 70.3% said they would buy fresh vegetables from a market vendor who had HIV/AIDS.

**Table 3:** Percentage of women with Accepting Attitude toward PLWHIV by their Socio-demographic variables

Variables		Willing to care a relative sick with HIV/AIDS “Yes” %	Would buy fresh vegetable from a market vendor who has HIV/AIDS “Yes” %	Would want HIV-positive female teacher who is not sick be allowed to continue teaching “Yes” %	Would want HIV infection in family remain secret “No”%	Have accepting attitude on all 4 indicators (“Yes” to the 1st 3 and “No” to the 4th) %
Overall score		86.2	70.3	71.3	36.6	20.6
*Age (years)*						
15–24		84.6	68.3	69.0	36.3	19.1
20–24		92.8	78.4	80.7	37.8	26.7
*Religion*						
Catholic		84.1	68.0	70.4	41.2	23.2
Protestant		88.3	73.7	73.6	35.5	20.5
Muslim		85.2	73.0	71.3	30.6	17.6
Pentecostal		88.3	64.7	68.3	33.0	17.1
Others		88.7	84.9	75.5	26.4	15.1
*Educational level*						
None		45.2	28.6	45.2	61.9	14.0
Primary		81.1	59.7	60.0	36.4	16.1
Secondary		93.9	84.7	85.8	35.4	26.3
Higher		97.7	89.2	90.8	35.9	28.5
*Place of residence*						
Urban		92.3	80.7	81.9	35.8	23.8
Rural		84.1	66.8	67.7	36.8	19.5
*Wealth index*						
Poorest		74.1	54.1	57.1	45.0	18.6
Poorer		79.4	64.4	64.6	38.2	20.5
Middle		84.5	64.0	68.4	37.7	19.4
Richer		86.5	72.7	69.5	33.8	18.9
Richest		93.8	79.8	81.4	34.6	23.1
*Ever been tested for HIV/AIDS*						
No		80.8	62.4	65.6	34.0	15.8
Yes		92.4	79.5	77.9	39.5	26.1
*Mass media (frequency of listening to radio/watching TV/reading newspaper or magazine)*						
Not at all		74.7	57.8	61.1	40.4	15.7
At least once a week		87.3	71.4	72.3	36.2	21.1

As shown in Table 4, young women who ever been tested for HIV and had access to mass media had higher knowledge of HIV/AIDS than those we had never been tested and without access to mass media, as reflected by the significant difference (59.8%, *P* < 0.001 and 53.2%, *P* < 0.01, respectively). Interestingly, young unmarried women who had ever been tested for HIV/AIDS were more likely to have a better comprehensive knowledge on HIV/AIDS as compared to their counterparts (OR 1.82, 95% CI 1.52–2.18 and aOR 1.37, 95% CI 1.12–1.67).

**Table 4:** Association of HIV/AIDS knowledge and socio-demographic characteristics

Independent variables	*N*^*a*^	HIV/AIDS comprehensive knowledge		X^2^ value	*P* value
		“Do not have”	“Have” ^b^		
*Age (years)*				27.77	< 0.001
15–19	1565	799(51.1%)	766(48.9%)		
20–24	390	141(36.2%)	249(63.8%)		
*Religion*				3.97	0.410
Catholic	772	387(50.1%)	385(49.9%)		
Protestant	605	278(46.0%)	327(54.0%)		
Muslim	244	109(44.7%)	135(55.3%)		
Pentecostal	282	141(50.0%)	141(50.0%)		
Others	53	26(49.1%)	27(50.9%)		
*Educational level*				134.37	< 0.001
No education	42	34(81.0%)	8(19.0%)		
Primary	1056	617(58.4%)	439(41.6%)		
Secondary	727	247(34.0%)	480(66.0%)		
Higher	130	42(32.3%)	88(67.7%)		
*Place of residence*				44.57	< 0.001
Urban	493	173(35.1%)	320(64.9%)		
Rural	1462	767(52.5%)	695(47.5%)		
*Wealth index*				72.04	< 0.001
Poorest	219	149(68.0%)	70(32.0%)		
Poorer	267	155(58.1%)	112(41.9%)		
Middle	341	169(49.6%)	172(50.4%)		
Richer	465	212(45.6%)	253(54.4%)		
Richest	662	254(38.4%)	408(61.6%)		
*Ever been tested for HIV/AIDS*				42.71	< 0.001
No	1044	574(55.0%)	470(45.0%)		
Yes	911	366(40.2%)	545(59.8%)		
*Mass media (frequency of listening to radio/watching TV/reading newspaper or magazine)*				13.04	< 0.001
Not at all	166	102(61.4%)	64(38.6%)		
At least once a week	1784	835(46.8%)	949(53.2%)		

^a^ Excluding missing values

^b^ “Have” HIV/AIDS comprehensive knowledge

In the present study, young women who had ever been tested for HIV exhibited a significantly better attitude towards PLWHIV compared to their counterparts (Table 5). Generally, respondents who obtained higher score on comprehensive knowledge about HIV/AIDS showed a tendency of positive accepting attitude towards PLWHIV. Again, after adjusting for potential confounding variables, the association remained positive as indicated in Table 6 (aOR 1.67, 95% CI 1.31–2.12).

**Table 5:** Association of acceptance attitude towards PLWHIV and socio-demographic characteristics

Independent variables	N^a^	Acceptance attitude towards PLWHIV		X^2^ value	*P* value
		“Do not have”	“Have”		
*Age (years)*				11.16	0.001
15–19	1567	1268(80.9%)	299(19.1%)		
20–24	389	285(73.3%)	104(26.7%)		
*Religion*				7.67	0.104
Catholic	772	593(76.8%)	179(23.2%)		
Protestant	604	480(79.5%)	124(20.5%)		
Muslim	245	202(82.4%)	43(17.6%)		
Pentecostal	281	233(82.9%)	48(17.1%)		
Others	53	45(84.9%)	8(15.1%)		
*Educational level*				33.77	< 0.001
No education	43	37(86.0%)	6(14.0%)		
Primary	1058	888(83.9%)	170(16.1%)		
Secondary	726	535(73.7%)	191(26.3%)		
Higher	130	93(71.5%)	37(28.5%)		
*Place of residence*				4.36	0.037
Urban	494	376(76.1%)	118(23.9%)		
Rural	1462	1177(80.5%)	285(19.5%)		
*Wealth index*				4.24	0.375
Poorest	220	179(81.4%)	41(18.6%)		
Poorer	268	213(79.5%)	55(20.5%)		
Middle	341	275(80.6%)	66(19.4%)		
Richer	466	378(81.1%)	88(18.9%)		
Richest	662	509(76.9%)	153(23.1%)		
*Ever been tested for HIV/AIDS*				31.78	< 0.001
No	1045	880(84.2%)	165(15.8%)		
Yes	911	673(73.9%)	238(26.1%)		
*Mass media (frequency of listening to radio/watching TV/reading newspaper or magazine)*				2.70	0.100
Not at all	166	140(84.3%)	26(15.7%)		
At least once a week	1786	1410(78.9%)	376(21.1%)		
*HIV/AIDS comprehensive knowledge*				33.56	< 0.001
“Do not have”	940	798(84.9%)	142(15.1%)		
“Have”	1015	754(74.3%)	261(25.7%)		

^a^ Excluding missing values

**Table 6:** Multivariable logistic regression analysis showing associations among HIV/AIDS knowledge, acceptance attitude and socio-demographic characteristics

Independent variables	HIV/AIDS comprehensive knowledge		acceptance attitude towards PLWHIV	
	OR (95%CI)	aOR (95%CI)	OR (95%CI)	aOR (95%CI)
*Age (years)*				
15–19	Ref	Ref	Ref	Ref
20–24	1.84(1.47–2.32)***	1.32(1.01–1.73)*	1.54(1.19–1.99)**	1.13(0.84–1.52)
*Educational level*				
No education	Ref	Ref	Ref	Ref
Primary	3.11(1.42–6.81)**	2.58(1.14–5.87)*	1.23(0.50–3.02)	1.25(0.49–3.22)
Secondary	8.47(3.85–18.66)***	5.51(2.39–12.69)***	2.30(0.94–5.65)	1.97(0.75–5.14)
Higher	9.18(3.90–21.65)***	4.20(1.69–10.44)**	2.53(0.97–6.62)	1.89(0.67–5.32)
*Place of residence*				
Rural	Ref	Ref	Ref	Ref
Urban	2.04(1.65–2.52)***	1.31(0.99–1.72)	1.30(1.01–1.65)*	0.98(0.71–1.35)
*Wealth index*				
Poorest	Ref	Ref	Ref	Ref
Poorer	1.54(1.06–2.23)*	1.16(0.78–1.72)	1.14(0.73–1.80)	0.92(0.57–1.49)
Middle	2.15(1.51–3.07)***	1.58(1.09–2.30)*	1.08(0.69–1.65)	0.82(0.52–1.30)
Richer	2.53(1.80–3.54)***	1.65(1.15–2.38)**	1.03(0.68–1.55)	0.72(0.46–1.13)
Richest	3.40(2.46–4.70)***	1.59(1.08–2.34)*	1.33(0.90–1.96)	0.77(0.48–1.23)
*Ever been tested for HIV/AIDS*				
No	Ref	Ref	Ref	Ref
Yes	1.82(1.52–2.18)***	1.37(1.12–1.67)**	1.89(1.51–2.36)***	1.56(1.23–1.98)***
*Mass media (frequency of listening to radio/watching TV/reading newspaper or magazine)*				
Not at all	Ref	Ref	Ref	Ref
At least once a week	1.83(1.32–2.54)***	1.33(0.94–1.88)	1.42(0.92–2.18)	1.19(0.75–1.86)
*HIV/AIDS comprehensive knowledge*				
“Do not have”	–	–	Ref	Ref
“Have”	–	–	1.96(1.56–2.45)***	1.67(1.31–2.12)***

^*^P < 0.05; **P < 0.01; ***P < 0.001, statistically significant difference; aOR = adjusted odds ratios

Comparative tests have shown statistically significant association between age group and knowledge on HIV/AIDS (Table 6). Women aged 20–24 years had better comprehensive knowledge on HIV/AIDS than those aged 15–19 years (OR 1.84, 95% CI 1.47–2.32). This association remained significant after adjustment and hence, women of 20–24 years were 32% more knowledgeable in terms of comprehensive knowledge about HIV/AIDS than the age group of 15–19 years (aOR 1.32, 95% CI 1.01–1.73). Similarly, there was a significant (*P* < 0.001) difference on HIV/AIDS comprehensive knowledge between urban and rural residents (Table 4). Women from urban residence were greater than twice more likely to know about HIV/AIDS comprehensive knowledge (OR 2.04, 95% CI 1.65–2.52) compared to those women from rural residence. However, the differences in place of residence and HIV/AIDS comprehensive knowledge were not significant after adjustment. The highest knowledge on HIV/AIDS was observed among women with higher educational levels. A statistically significant associations were found across all educational levels as compared to women who did not attend education at all (Table 4). As educational status of the respondents increased, so did the strength of association, indicating the highest association in women with higher educational status (OR = 9.18; 95% CI 3.90–21.65). After adjustment, the association remained significant (Table 6). In this study, we found significant association between higher household wealth status and greater comprehensive knowledge on HIV/AIDS (*P* < 0.001) (Table 4).

In the logistic regression analysis (Table 6), women aged 20–24 years had higher odds (OR 1.54; 95% CI 1.19–1.99) of having accepting attitude towards PLWHIV compared with adolescent women of 15–19 years old. However, this association was not significant in multivariable logistic regression analysis. Young women residing in urban areas had more positive attitude towards PLWHIV than those rural areas (*P* < 0.01) indicating an independent significant positive association. Nevertheless, this association became insignificant after adjusting the OR in multivariable logistic regression analysis.

## Discussion

Youth population, the major human capital of any society, should be well informed and evaluated on comprehensive knowledge about HIV/AIDS and attitude towards PLWHIV. In this study, almost all (99.3%) of young Ugandan women were found to be aware of HIV/AIDS. This is consistent with the results of the Uganda AIDS Indicator Survey (UAIDS-2011) [29]. However, only 51.9% have comprehensive knowledge on HIV/AIDS, which is contrary to the United Nations General Assembly’s (UNGASS) 2001 plan on improving HIV/AIDS knowledge among youth. The UNGASS target was to provide access on accurate information and good service to 95% of individuals aged 15–24 years worldwide by 2010 [30]. Corresponding to the present study, Uganda HIV/AIDS progress report stated that “many people are vulnerable to infection due to lack of comprehensive knowledge on HIV/AIDS as well as local misconceptions surrounding the HIV/AIDS phenomenon” [9]. Similar studies from Africa and Asia noted that comprehensive knowledge of HIV/AIDS is inadequate [14, 28, 31]. Nevertheless, studies from Europe showed a high level of HIV/AIDS-related Knowledge [32]. Participants’ inadequate knowledge about HIV/AIDS can account for inadequate access to sexual and reproductive health information [32]. Gender inequalities and dangerous traditional norms concerning issues of sexual health and sexuality could also account for the low levels of knowledge among young women [21, 33].

Methods of reducing HIV transmission include but are not limited to, loyalty to one uninfected partner and consistent use of condoms during intercourse. In this study, participants’ knowledge about prevention methods of HIV/AIDS transmission is moderately high, and hence there is still a room for improvement. Young women’s knowledge of prevention methods (condom use and having a faithful uninfected partner) was fairly high at (70.4%). These findings are similar to the reports of UDHS-2006 and UAIS-2011 [29, 34]. However, similar studies from Iran and Ethiopia showed a substantial gap in knowledge. Only 49.8% and 52% of young women were aware of the methods for prevention of HIV transmission, correspondingly [14, 35]. To determine effective techniques for HIV/AIDS prevention and eradicate popular misconceptions, it is useful to identify fallacies about HIV/AIDS [29]. Two-third (66.9%) of the respondents rejected the misconception that HIV can be transmitted through mosquito bites. About 13% believed the possibility of transmission by witchcraft or other supernatural, and 19.5% by sharing a meal with someone who is infected with HIV. The misconceptions reported in this study could exacerbate risky sexual behaviors, which may predispose them to HIV infection [36]. The findings further pointed out the need to strengthen educational activities by clarifying the puzzles about knowledge on HIV transmission and relevant misconceptions. Having adequate comprehensive knowledge about HIV/AIDS can also reflect young women’s behavior. As a consequence, they may avoid harmful practices thereby safeguarding themselves from HIV/AIDS [36].

Data from multivariable logistic regression analysis demonstrated that higher educational status of young women was significantly associated with having comprehensive knowledge about HIV/AIDS. We found out that respondents with primary, secondary and higher levels of education had more comprehensive knowledge regarding HIV/AIDS compared to those with no education at all. In line with the current findings, previous studies [14, 28, 37] demonstrated beyond doubt that education is a social vaccine and is a critical component in the fight against HIV/AIDS. Based on these findings, there is a need to provide extracurricular activities on HIV/AIDS for the young age groups. Expectedly, the current study found a higher level of knowledge among the inhabitants of urban areas than rural areas. This result is consistent with the findings from previous studies [13, 28, 38]. Moreover, a DHS survey conducted in eight settings in SSA (Uganda, Malawi, Kenya, Lesotho, Tanzania, Zambia, Swaziland, and Zimbabwe) with high HIV prevalence reported that knowledge on HIV/AIDS was higher among urban residents [39]. This could be elucidated by an uneven distribution of access to information through the media and instructional campaigns across various regions [32]. In addition, wealthier women were more knowledgeable compared to their counterparts. This could be due to their high living standards allowing them to get more access to education which in turn boosts their knowledge about HIV/AIDS [13]. In harmony with several previous studies [13, 14, 28], our study demonstrated higher odds of comprehensive knowledge among “richest”, “richer” and “middle” wealth category than the “poorest”.

Consistent with a study done in Kenya [40], despite extensive exposure to mass media (91.5%), considerable proportions of young unmarried women showed inadequate knowledge regarding HIV/AIDS. The current study revealed that women who had ever been tested for HIV/AIDS were more likely to have adequate knowledge on HIV/AIDS. This could be the result of education and counselling received from healthcare providers.

Negative attitude towards PLWHIV is common worldwide [21] and is regarded as a fundamental barrier in the struggle against HIV/AIDS [28]. However, efforts directed at lowering negative attitude rank low in the priority list of HIV/AIDS programs [16]. The spread of HIV/AIDS has generated anxiety, fear as well as prejudice to PLWHIV [29]. In Uganda, although efforts have been made to reduce the negative attitude towards PLWHIV [8], the lack of well-defined social support package and capacity gaps in traditional institutions have stymied the national response. Consequently, stigma towards PLWHIV remains high [9, 29].

Regarding attitudes towards PLWHIV, only 20.6% of the participants had a positive acceptance attitude to PLWHIV. Comparable population-based studies conducted in the Democratic Republic of Congo (DRC), Nigeria, Iran, and India also found a high level of stigma ranging from 3.2 to 37% [28, 32, 41]. In concordance with an Iranian study [32], majority (86.2%) exhibited willingness to care for a relative with HIV. Approximately 36% of young unmarried women reported that they would not want HIV infection in a family to remain secret. This low rate of disclosure shows negative attitude is becoming a major concern in tackling the burden of HIV/AIDS. Nearly 30% of the participants had negative beliefs toward buying fresh vegetables from a vendor with HIV or attending a lecture taught by a female HIV-positive teacher. This discriminatory attitude on PLWHIV could be a barrier to voluntary counselling and testing for HIV and the productive dissemination of enlightenment programs [36]. The high level of stigma uncovered in this study might be attributed to the inadequate knowledge about preventing HIV transmission and fallacies regarding HIV/AIDS [32, 42, 43]. Similar studies done in Nigeria, India, Kenya, and Burundi [21, 28, 44, 45] noted that women aged 20–24 years had a better attitude towards PLWHA than those aged 15–19 years. In this study, young women aged 20–24 years were 1.54 times more likely to have a better attitude towards PLWHIV than those aged 15–19 years. However, no significant association was seen after adjusting for age in multivariable logistic regression. Effective and efficient educational instruction attained from educational institutions about HIV/AIDS can change one’s negative attitude [28, 46] and may subsequently encourage healthy behaviors [14]. In addition to this, young women who are not in school might lose the chance of obtaining knowledge about HIV/AIDS [13]. In the current study, no association was found between educational status and a positive attitude towards PLWHIV. Easy access to health-related information or services from mass media by urban residents could help them acquire better knowledge about HIV/AIDS and accepting attitude than rural residents [21]. However, in this research, the variation in accepting attitude among the different places of residence (rural and urban) as well as access to mass media (no access at all and once a week) were insignificant. This finding is contrary to UAIS-2011 report [29] and other studies [14, 32, 47–49]. Besides, many other studies have demonstrated that wealth status is significantly associated with positive attitude towards PLWHIV. Although the pattern among the four wealth quartiles was not uniform, previous studies had reported that those with higher socio-economic status were more likely to have accepting attitude towards PLWHIV than those with lower socio-economic status [21, 28, 50, 51]. Contrary to that assumption, wealth status was not significantly associated with accepting attitude towards PLWHIV in the bivariate analysis. Since economic solvency assures access to indispensable necessities such as standard of living, healthcare services, and education; the absence of relationship between wealth status and positive attitude towards PLWHIV is hard to explain. Indeed, additional studies are warranted in the future with regard to this issue.

As indicated in various studies, knowledge has been identified as a major driver of positive attitude towards PLWHIV. On the other hand, negative attitudes appear to be the outcome of poor access to appropriate and accurate HIV/AIDS-related information [52]. However, knowledge may occasionally stem risky sexual behaviors [14]. In this regard, our findings revealed that those with comprehensive knowledge on HIV/AIDS were highly likely to have accepting attitude compared to the reference group. This finding is in agreement with many similar studies existing in the literature [37, 42, 43, 48, 53]. The health education sessions provided by health experts, especially those visiting HIV/AIDS testing centers could have a positive effect on attitude towards PLWHIV. In this study, women who ever had HIV/AIDS testing were more likely to show positive attitudes towards PLWHIV.

### Limitation

In this study, we have noted some limitations. Firstly, since DHS is a household survey, the exclusion of people living in institutions or on the street might limit the generalizability of the results. Secondly, we failed to report cause and effect due to the cross-sectional nature of the study. Thus, our results can be explained referring to observed associations between explanatory and outcome variables. Thirdly, this kind of study can be affected by recall bias which might affect the findings. Moreover, the research participants could fail to express their real feelings and attitudes in a face-to-face interview which could affect the quality of the data. Finally, we could not use complex sampling procedure due to the fact that one of the elements (sample strata for sampling errors V022) was unavailable.

## Conclusion

Our study demonstrated a remarkably high level of HIV/AIDS awareness in the participants. However, a considerable gap in knowledge about HIV/AIDS and a high level of stigma towards PLWHIV was uncovered among young unmarried women in Uganda. Urban residency, age (being younger), higher level of education, higher socio-economic status, previous HIV test were associated with adequate comprehensive knowledge. Moreover, previous HIV test, secondary educational level and comprehensive knowledge about HIV/AIDS, were significantly associated with a positive attitude towards PLWHIV. Importantly, we have to note that the reported results may derail the country’s quest to meet UNAIDS target of ending the AIDS epidemic as well as reducing new HIV infection to fewer than 100,000 by 2020 among adolescents and young people. Therefore, expanding access to comprehensive sexuality education, especially to those in the lower ladder of socioeconomic status, rural residents, and uneducated should be prioritized. Finally, further research on the connection between knowledge on HIV/AIDS and risky sexual behavior is warranted in the future.

## Data Availability

Relevant data are displayed in this article and upon reasonable request, additional data might be sought from the corresponding author. The raw data we extracted and used for our study can be accessed by visiting the following website: https://www.dhsprogram.com/data/model-datasets.cfm

## Abbreviations

PLWHIV: People Living with Human Immunodeficiency Virus
UDHS: Uganda Demographic Health Survey
UAIS: Uganda AIDS Indicator Survey
